# Analysis of exosome-derived microRNAs as early biomarkers of lipopolysaccharide-induced acute kidney injury in rats

**DOI:** 10.3389/fphys.2022.944864

**Published:** 2022-08-26

**Authors:** Carolina Carvalho Serres Da-Silva, Ana Carolina Anauate, Tatiana Pinotti Guirao, Antônio da Silva Novaes, Edgar Maquigussa, Mirian Aparecida Boim

**Affiliations:** ^1^ Renal Division, Department of Medicine, Universidade Federal de SP, São Paulo, Brazil; ^2^ Postgraduate Program of Health and Environment, Universidade Metropolitana de Santos, Santos, Brazil

**Keywords:** acute kidney injure, sepsis, miRNA, microRNA, exosome (vesicle), lipopolyssacharide

## Abstract

Sepsis contributes to the high prevalence of acute kidney injury (AKI), which mainly occurs in hospitalized patients. The delay in AKI detection is a risk factor for death and chronicity; thus, early diagnosis is essential for initiating proper treatment strategies. Although serum creatinine is used as biomarker, it is increased in plasma serum creatinine only at late stages of AKI. MicroRNAs (miRNAs), a class of noncoding RNAs responsible for gene regulation, can be found in biological fluids within vesicles such as exosomes and may be promising tools for the early detection of AKI. We aimed to identify potential blood miRNAs that can be used as early biomarkers of sepsis-induced AKI in rats. Adult male Wistar rats received a single dose of lipopolysaccharide (LPS). The earliest significant increase in serum creatinine was detected 4 h after LPS administration. To evaluate whether miRNAs could act as early biomarkers, blood samples were collected before and 2 h after LPS infusion. Serum NGAL levels were used as a comparative marker. Serum miRNAs were derived from exosomes, and their expression were evaluated by the PCR array. *miR-181a-5p* and *miR-23b-3p* showed higher expression in LPS-treated rats than in the control animals (*p* < 0.05). Bioinformatics analysis showed that both miRNAs target molecules associated with transcription factors that regulate genes related to proinflammatory cytokines. Considering that LPS activates transcription factors that lead to the production of proinflammatory cytokines, possible premature changes in the serum levels of *miR-181a-5p* and *miR-23b-3p* may be used to identify sepsis-induced AKI earlier.

## 1 Introduction

Acute kidney injury (AKI) is a clinical syndrome that occurs in approximately one-third of hospitalized patients and is associated with high morbidity and mortality (Bellomo et al., 2004). Currently, sepsis is the most common cause of AKI in hospitalized patients. Septic patients who develop AKI have a worse prognosis and an increased risk of chronic kidney disease (CKD) and death (Schrier and Wang, 2004; Lo et al., 2009; Levey and James, 2017). The pathophysiology of sepsis-induced AKI can occur through hemodynamic and nonhemodynamic mechanisms; intrarenal vasoconstriction, altered blood flow distribution, tissue toxicity and ischemic changes are the most frequent mechanisms (Ma et al., 2019).

AKI is diagnosed by decreased urine output and increased serum creatinine levels (Khwaja, 2012); however, serum creatinine, which is currently the most frequently used marker of the glomerular filtration rate (GFR), can be influenced by other variables that are unrelated to renal damage, such as age, race, nutritional status, muscle mass, enteral/parenteral diets, catabolic status and volume status (Thomas et al., 2015). In addition, the increase in serum creatinine is detectable when renal damage is at a more advanced stage and there is greater impairment of renal function, which in turn makes recovery more difficult. Therefore, it is important to find new, more sensitive and earlier markers of renal injury, which would allow real-time follow-up and predict the risk of developing AKI and/or the risk of progression to more advanced stages.

In recent years, several biomarkers have been considered to detect AKI at an early stage. Among them, the 25 kDa protein neutrophil gelatinase-associated lipocalin (NGAL) stands out. In fact, plasma NGAL concentrations are increased in sepsis (Mårtensson et al., 2010); however, changes in NGAL levels are not exclusive to renal injury and can be high in acute infectious conditions, such as pancreatitis, heart failure and cancer, even with no signs of AKI (Hjortrup et al., 2013).

Thus, microRNAs (miRNAs), a class of small, noncoding RNAs, stand out as potential biomarkers for the early diagnosis of AKI. miRNAs are single-stranded RNAs consisting of approximately 22 nucleotides (Ha and Kim, 2014) that can be totally or partially complementary to the 3′ untranslated region (3′UTR) of target messenger RNAs (Bhatt et al., 2016). Through this binding, miRNAs regulate gene expression by degrading or impeding target mRNA translation. Thus, miRNAs suppress the translation of mRNAs, reducing protein synthesis. In addition, because of its short length, a single miRNA is able to regulate the expression of many mRNAs (Simpson et al., 2016).

The recognition of miRNAs as key factors in cellular physiology and pathophysiology is well established. miRNAs are secreted by cells through microvesicles, including exosomes, and they remain stable in body fluids such as blood, saliva, urine, and feces. (Hu et al., 2010; Gallo et al., 2012; Lv et al., 2013). Several miRNAs have been previously associated with the pathophysiology of AKI of many etiologies (Bhatt et al., 2016; Fan et al., 2016; Liu et al., 2016; Zou and Zhang, 2018), including septic AKI. Because sepsis-induced AKI can alter the expression levels of specific miRNAs, these molecules may potentially be effective tools for the early detection of sepsis-induced renal injury; however, to our knowledge, there are no studies in the literature analyzing the early miRNA profile in sepsis. In the present study, we developed a rodent model of sepsis-induced AKI to identify early miRNAs that were differentially expressed in the serum exosomes of septic animals.

## 2 Materials and methods

### 2.1 Rodent model of sepsis-induced acute kidney injury and serum creatinine analysis

All experimental procedures were approved by the Ethics in Research Committee of the Federal University of Sao Paulo (CEUA-UNIFESP #3083130317). Male Wistar rats weighing 150–200 g were used to establish the sepsis-induced AKI model. Animals were purchased from the animal facility of the Federal University of Sao Paulo, Brazil and were housed in collective cages (5 animals/cage) at room temperature with a 12 h light/dark cycle and free access to standard food and tap water. The basal parameters were obtained from blood sampled by venipuncture of the retro-orbital sinus under sedation. After 15–21 days, the animals received 7.5 mg/kg LPS from *Escherichia coli* (strain 0111:B4) intraperitoneally (i.p.). Pilot experiments were performed to determine the ideal dose of LPS that was enough to induce AKI, and 7.5 mg/kg was determined to the most suitable dose.

First, the animals were divided into 4 groups containing six animals in each group to determine the kinetics of the increases in serum creatinine. Animals received 7.5 mg/kg LPS, and blood samples were collected at different times (2 hr, 4 hr, 6 hr, and 8 h) after LPS administration. After this experiment, another group of animals (n=5) received LPS (7.5 mg/kg). The animals were anesthetized with xylazine/ketamine (5 mg/kg/75 mg/kg), and blood samples were collected before (basal) and 2 h after LPS administration. Animals were euthanized by anesthetic overdose. All blood samples were immediately centrifuged, and the serum was stored at -80 °C until use.

Serum creatinine concentrations were determined by Jaffe’s method. Serum concentrations of NGAL were measured using the *rat NGAL ELISA kit* (BioPorto, Denmark) according to the manufacturer’s instructions.

### 2.2 Exosome-derived RNA extraction, cDNA synthesis and preamplification of microRNAs

Total RNA, including miRNAs, was isolated from serum exosomes using the commercial exoRNeasy serum plasma midi kit (Qiagen, Germany) according to the manufacturer’s instructions. Exogenous *cel-miR-39-3p* (Qiagen) was added to the samples to measure the RNA isolation efficiency, as determined by the manufacturer.

The complementary DNA (cDNA) was then synthesized from 100 ng of total RNA by using the miScript^®^ II RT kit (Qiagen), and miRNA preamplification was performed using the miScript^®^ PreAmp PCR kit (Qiagen) according to the manufacturer’s instructions.

### 2.3 RT-qPCR array to analyze miRNA expression

The expression levels of each miRNA were determined by RT-qPCR in 5 serum samples from control (basal) animals and 5 serum samples from animals 2 h after LPS administration. PCR arrays were performed using the QuantiStudio 7 system (Life Technologies, EUA) according to the manufacturer’s instructions. The expression levels of the miRNAs were normalized to the *cel-miRNA-39-3p*, *SNORD61*, *SNORD68*, *SNORD95*, *SNORD96A* and *RNU6-2* controls using *Data Analysis Center software* (Qiagen).

### 2.4 Target genes and pathway analysis of the dysregulated microRNAs

The miRNA target gene databases miRDB (http://mirdb.org/) and TargetScan (http://www.targetscan.org/) were used to predict the target genes of the differentially expressed miRNAs. miRDB is an online bioinformatics tool for predicting molecular targets of microRNAs (Wong and Wang, 2014). TargetScan is another online bioinformatics tool for predicting biological targets of miRNAs that searches for the presence of conserved sites that can bind with the seed region of each miRNA (Lewis et al., 2005).

The identified target gene set and the Enrichr database (http://amp.pharm.mssm.edu/Enrichr/) were used to analyze the biological process, molecular function, cellular component and pathways that were significantly enriched by the target genes. This online software integrates several databases and, from the target genes listed, is able to identify enriched transcription factors associated with these genes (Chen et al., 2013).

### 2.5 Analysis of exosome-enriched extracellular vesicles

The exosome size and concentration were determined using a Malvern Nanosight Tracking Analysis (NTA) system (NS300) (Worcestershire, United Kingdom). All samples were diluted according to the limit capacity of the equipment and the particle concentration (particles/mL) was calculated.

### 2.6 Statistical analysis

The paired *t-*test was used to compare mean creatinine levels between the LPS-treated animals and the respective basal values. The Shapiro-Wilk test was used to assess the distribution of NGAL, which did not show a normal distribution. Therefore, the Wilcoxon test was used to compare the NGAL levels in the serum of the animals 2 h after LPS injection compared to basal levels. The data were considered statistically significant when *p* < 0.05.

## 3 Results

### 3.1 Characterization of the rodent sepsis-induced AKI model

**FIGURE 1:**
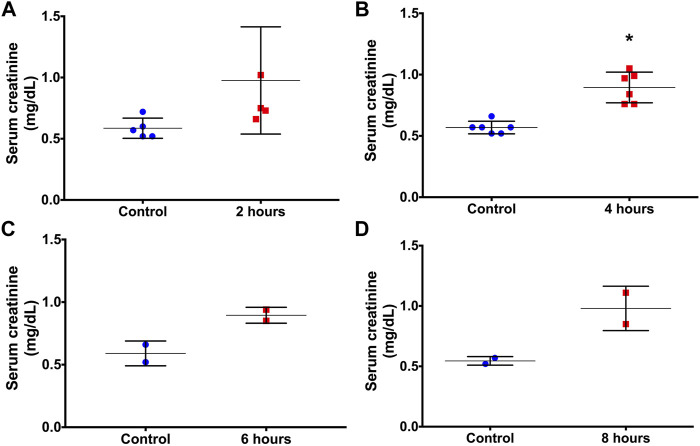
Serum creatinine concentrations before (controls) and at 2 **(A)**, 4 **(B)**, 6 **(C)** and 8 **(D)** hours after LPS administration. *p < 0.05 vs the control (paired *t-*test).

Results are presented as mean±standard deviation. High lethality was observed mainly after more prolonged period after LPS administration. One animal died after 2 h, and 4 animals died after 6 and 8 h. As shown in Figure 1, there was an increase in serum creatinine 2 h after LPS administration, but the difference was not significant. Serum creatinine was significantly increased at 4 h, demonstrating that the detection of renal dysfunction by serum creatinine levels was possible at 4 h after LPS administration (Figure 1).

### 3.2 Sepsis-induced AKI induces the release of small exosomes in the serum

**FIGURE 2:**
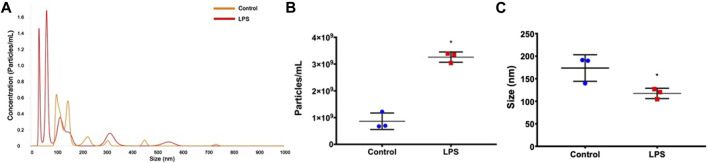
Exosome characterization. **(A)** Histogram representing the profile of nanoparticle size by nanoparticle tracking analysis. **(B)** Exosome concentration. **(C)** Exosome size. *p < 0.05 vs the controls (paired *t*-test).

Initially, we examined whether LPS stimulation interfered with the release of exosomes. The representative image of size distribution and particle concentration of exosomes between two groups is shown in Figure 2A. Increased exosome release was observed in LPS-stimulated animals compared to basal control (*p*=0.005; Figure 2B). In addition, a significantly smaller exosome size was observed in the LPS treatment group than in the control group (*p*=0.036; Figure 2C). These data indicate that LPS influences both the release and size of circulating extracellular vesicles in rats.

### 3.3 Analysis of miRNA profiles

To identify an earlier biomarker of sepsis-induced AKI than creatinine, the expression profile of miRNAs in samples collected 2 h after LPS administration, which was an earlier time than that needed to detect serum creatinine elevation, was evaluated.

**FIGURE 3:**
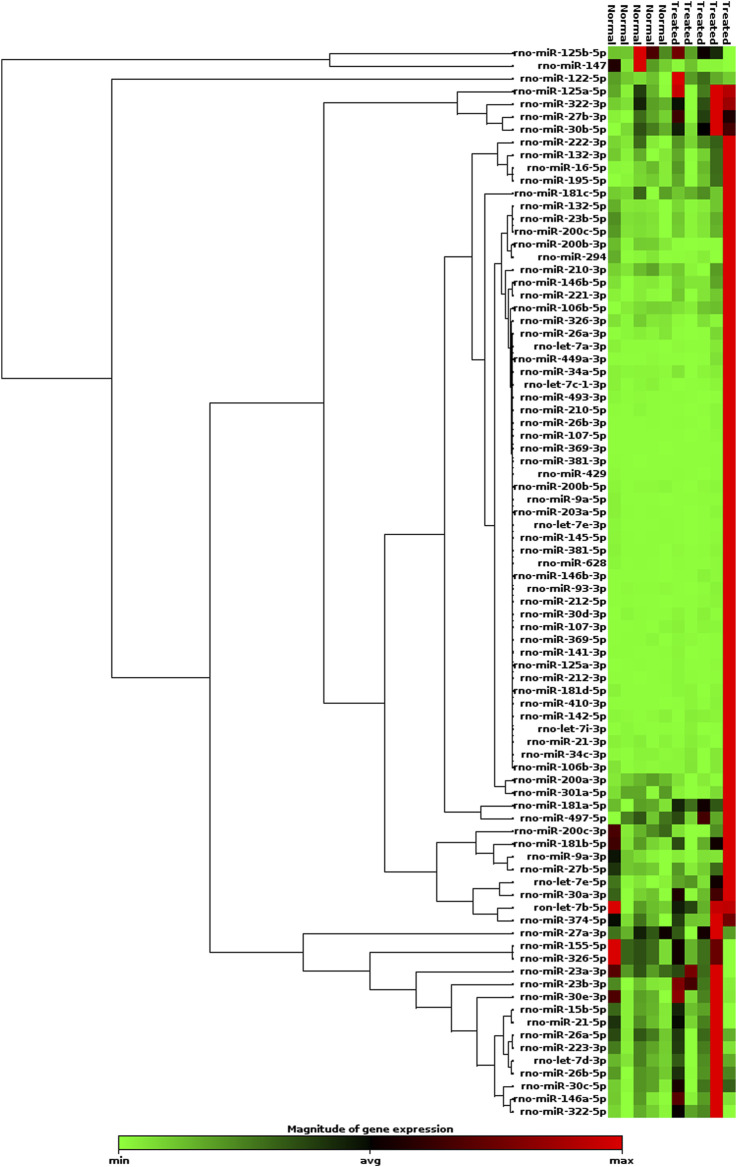
Heatmap of the expression of 84 miRNAs evaluated by RT-qPCR array. LPS (*n*=5); Control (*n*=5). Red indicates high relative expression, and green denotes low relative expression.

**FIGURE 4:**
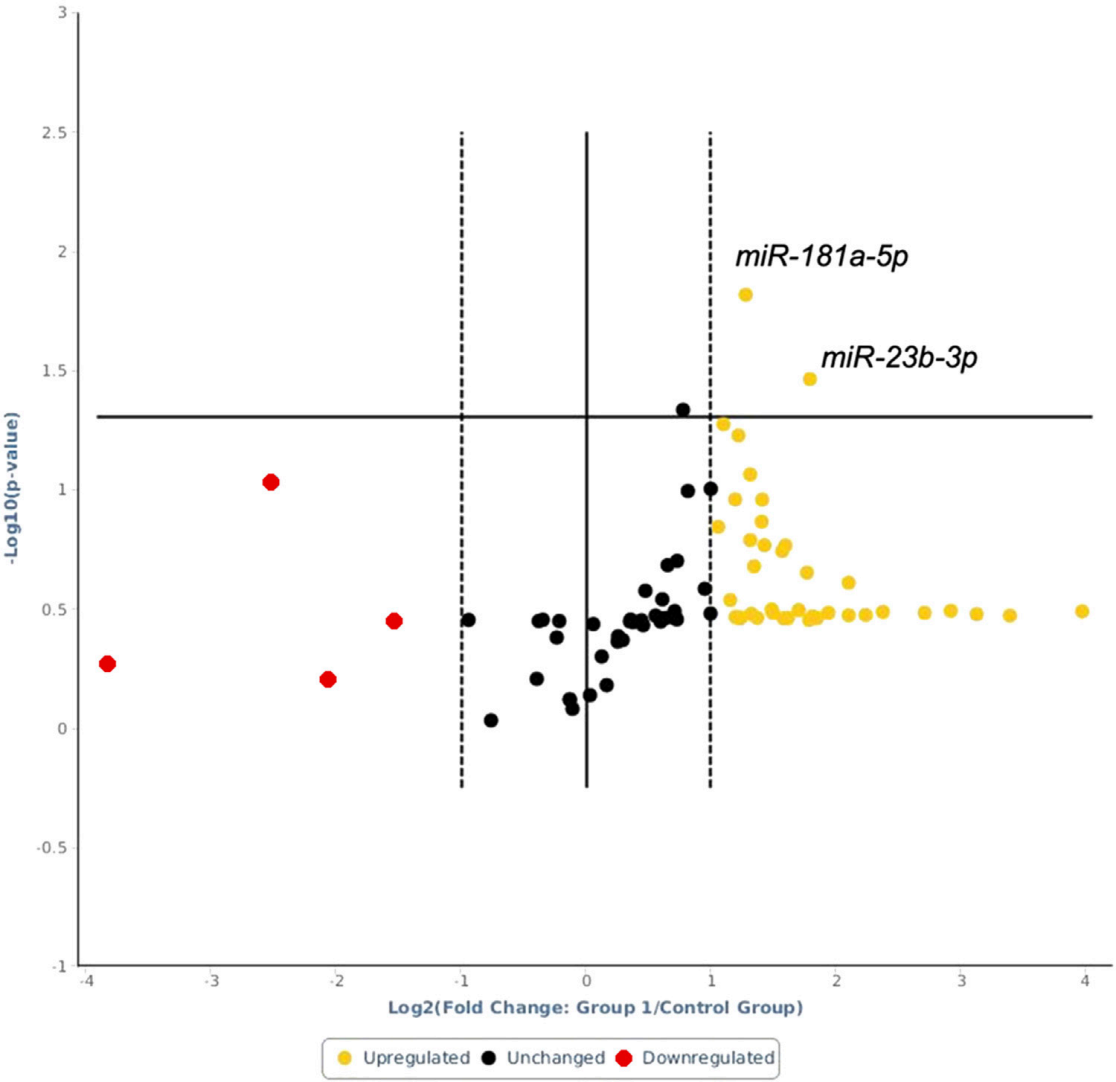
Volcano plot of the analyzed miRNAs. Above the horizontal line are the differentially expressed miRNAs with statistical significance in LPS-treated animals relative to the controls. The *x*-axis shows log 2 (fold change) changes between the LPS-treated animals and the control animals, while the *y*-axis shows the log-10 of the *p* value.

The expression heatmap of the miRNAs identified in exosomes isolated from the serum of control or LPS-treated animals is shown in Figure 3. The PCR array results showed that of the 84 miRNAs identified, 40 had increased expression and 4 had decreased expression (Supplementary Table S1) compared to basal control levels. Only 2 upregulated miRNAs were statistically significant, *miR-181a-5p* and *miR-23b-3p,* and these miRNAs exhibited increased expression after LPS treatment compared to those of controls (*p*=0.015 and *p*=0.035, respectively; Figure 4). Downregulated miRNAs were not statistically significant.

### 3.4 Analysis of *miR-181a-5p* target genes

Using the miRDB and TargetScan bioinformatics tools, we verified the target genes of *miR-181a-5p*. Of these target genes, we selected the 10 targets with potential involvement in the pathophysiology of AKI, as shown in Supplementary Table S1. We found that only the *ZNF594* gene was identified as a target gene of *miR-181a-5p* by the two tools. This gene encodes a “zinc finger” nuclear protein that is capable of binding to DNA and is related to mechanisms that control gene transcription (Jen and Wang, 2016).

To better understand the biological role of *miR-181a-5p*, we used the Enricher platform. As shown in Supplementary Table S2, according to this tool, *miR-181a-5p* is associated with many physiological processes, including the regulation of transcription, regulation of macromolecule synthesis and regulation of gene expression. Analysis by Enricher demonstrated that *miR-181a-5p* mediates these mechanisms by interfering with DNA dynamics, as shown in Supplementary Table S3, and is associated with several organelle functions (Supplementary Table S4) and pathophysiological processes, including prostate cancer (Supplementary Table S5). These results suggest that *miR-181a-5p* has several cellular functions, from gene transcription to vesicle formation.

### 3.5 Analysis of *miR-23b-3p* target genes

*miR-23b-3p* and the top 10 target genes are shown in Supplementary Table S1. The two target genes *AUH* and *FAM234B* were identified by the two tools used in this investigation. The Enricher platform identified the biological processes (Supplementary Table S2), molecular functions (Supplementary Table S3), cell components (Supplementary Table S4) and pathways (Supplementary Table S5) associated with *miR-23b-3p*. Among the many biological processes regulated by *miR-23b-3p,* the cellular response to drugs (Supplementary Table S2) is of interest since it could have an impact on the results obtained in this study. This activity may be related to the role of *miR-23b-3p* in upregulating 3′,5′-cyclic AMP and cyclic 3′,5′-nucleotide phosphodiesterase activity (Supplementary Table S3).

### 3.6 Serum NGAL is increased in the rodent sepsis-induced AKI model

**FIGURE 5:**
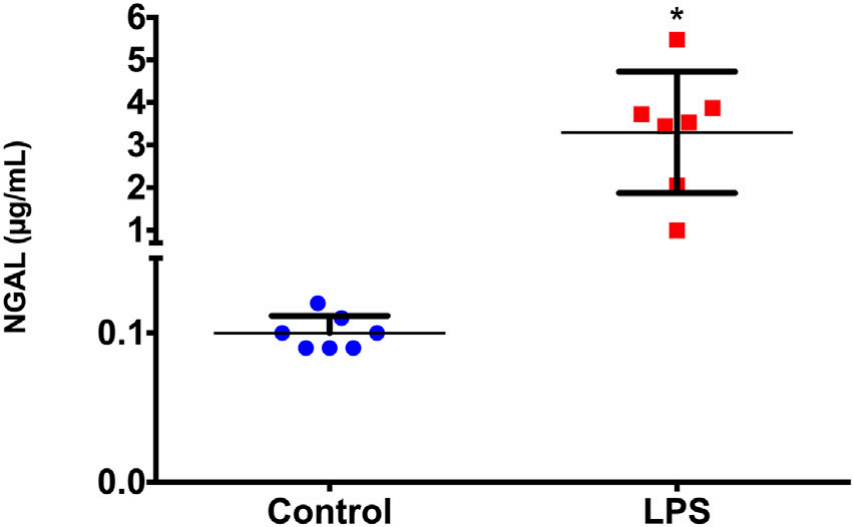
Serum NGAL concentrations before (basal) and 2 h after LPS administration. *p < 0.05 vs basal levels (Wilcoxon test).

As a possible tool to validate the results obtained, we used the NGAL serum concentration, which was measured in samples of the animals 2 h after LPS injection to verify whether this marker was also altered. We observed that NGAL showed a statistically significant increase (*p*=0.018) in serum samples from animals 2 h after LPS injection compared to those of the controls (Figure 5).

## 4 Discussion

Sepsis is one of the most common causes of AKI in clinical practice, is characterized by very high mortality, and mainly occurs in hospitalized patients. The use of early therapeutic strategies, such as dialysis, may help reduce the morbidity and mortality caused by the clinical consequences of sepsis-induced AKI. Identifying the molecules and pathways involved in sepsis-induced AKI is extremely important to better understand the development of this disease, as well as to identify sensible biomarkers capable of predicting the decline in renal function at an earlier stage and, consequently, enabling appropriate therapeutic strategies at the most opportune moments. The identification of urinary markers can be difficult because of the reduced urine output that is typical of most forms of AKI. In addition, several studies have identified miRNAs associated with sepsis-induced AKI (Leelahavanichkul et al., 2015; Colbert et al., 2017; Fu et al., 2017; Ge et al., 2017; Chen Y. et al., 2018; Zheng et al., 2018; Yongjun et al., 2019); however, most of these studies identified tissue miRNAs that would make them unlikely to be AKI biomarkers in clinical practice. miRNAs transported by extracellular vesicles present in the serum are easily obtained with minimal invasiveness and can therefore be used as biomarkers of sepsis-induced AKI. In the present study, two differentially expressed miRNAs were isolated from circulating extracellular vesicles and identified in this sepsis-induced AKI model: *miR-181a-5p* and *miR-23b-3p*. Both miRNAs were upregulated before the increase in serum creatinine and therefore at an earlier stage of sepsis-induced AKI development.

*miR-181a-5p* has been reported to be involved in many pathophysiological mechanisms associated with different types of cancer, (Yu C. et al., 2019; Yu J. et al., 2019; Mao et al., 2019; Zhu et al., 2019), hypertension (Marques Francine et al., 2011; Marques et al., 2015; Mathe et al., 2018), diabetic nephropathy and renal fibrosis (Xu et al., 2017). Interestingly, *miR-181a-5p* was increased in extracellular vesicles in the plasma of septic mice (Xu et al., 2018) and induced proinflammatory cytokines such as MIP-2, IL-6, IL-β, TNF-α and FB (Xu et al., 2018). Moreover, it has been demonstrated that *miR-181a* also interferes with the expression of IL-8 by modulating the immune receptor TLR4 (Galicia et al., 2014). Sepsis is characterized by severe inflammatory responses; thus, these results suggest that *miR-181a-5p* may constitute a very early signal of inflammation in response to sepsis, and its increase in plasma can be detected before serum creatinine elevation. The increase in *miR-181a-5p* before renal function weakening suggests that *miR-181a-5p* is a potential biomarker of sepsis-induced AKI; however, its association with the decline in renal function in humans needs additional study.

We also observed upregulation of *miR-23b-3p*, and similar to *miR-181a-5p*, this miRNA is also associated with several types of neoplasms (Wei et al., 2018; Xian et al., 2018; Yang et al., 2018; Moreno et al., 2019; Rezaei et al., 2019). Interestingly, the presence of *miR-23b-3p* in human urinary exosomes has been reported, and its expression was increased in urinary exosomes of patients with nephrotic syndrome (Cheng et al., 2014). There is evidence that *miR-23b-3p* regulates multiple cellular processes in podocytes, and therefore, increased *miR-23b-3p* expression may be a consequence of glomerular and podocyte damage (Chen T. et al., 2018). Similar to *miR-181a-5p, miR-23b-3p* can regulate inflammatory responses (Smith et al., 2003), including the primary T cell immune response (O'Connor et al., 2008). Other aspects related to *miR-23b-3p* that are relevant in the context of the present study include the regulation of the protein PPARGC1A, which participates in the regulation of nephron segmentation during embryogenesis (Chambers et al., 2018). Additionally, PPARGC1A deficiency was associated with renal inflammation and increased nephrotoxic severity in AKI (tubular cell death and compensatory proliferation of these cells) (Fontecha-Barriuso et al., 2019). Thus, these results, together with those of the present study, reinforce that *miR-23b-3p* targets important proteins in renal inflammation related to sepsis-induced AKI.

The increase in *miR-181a-5p* and *miR-23b-3p* expression 2 h after LPS administration was coincident with the increase in NGAL in the serum of LPS-treated animals. Although NGAL has been indicated as a gold standard biomarker for many forms of AKI, it is important to emphasize that the diagnostic capacity of NGAL remains somewhat controversial, especially in the context of AKI. NGAL is a marker of minimal tubular damage, especially in sepsis (de Geus et al., 2013; Macdonald et al., 2017); however, there is no evidence the accuracy of NGAL measurement in predicting sepsis-induced AKI (de Geus et al., 2013). On the other hand, the increase in serum NGAL 2 h after LPS may validate our results and indicate that the miRNAs identified in the present study can be used as potential early biomarkers of LPS-induced renal damage.

Finally, our results showed that LPS induced an increase in the number of extracellular vesicles in serum. Based on size, these vesicles were mainly exosomes. This result was consistent with those of other studies showing that pathogen infection can stimulate exosome and proinflammatory cytokine release as a mechanism of immune surveillance (Bhatnagar et al., 2007; Bhatnagar and Schorey, 2007; Essandoh et al., 2015). We observed that extracellular vesicles are significantly affected by LPS by decreasing the vesicle size and by regulating the vesicular miRNA content. Exosomes ranged from 30 to 150 nm in size (Chen et al., 2022). The size of the exosomes control group showed an average of 174 nm. However, the highest concentration peaks in the control group are smaller than 150 nm in diameter (Figure 2A). One factor indicating increased exosome size is aggregates of vesicles that can be interpreted as a single vesicle by NTA and showed a larger vesicle size. This data was observed by Novaes et al. in a study that showed aggregates with variable sizes of exosomes by electron microscopy (da Silva Novaes et al., 2019). Our results revealed a significant decrease in the size of the exosome in the LPS group (Figure 2C). Bell et al. examined the size exosome characteristics of AC16 human cardiomyocytes stimulated with LPS. The results revealed that LPS significantly decreased mean exosome size (Bell et al., 2019). These findings corroborate that size of the exosomes decreases substantially in cells stimulated with LPS. Other stimuli also demonstrated similar results in a reduction of the size of exosomes, like extracellular osmotic stress (Fathali et al., 2017), sonication (Nizamudeen et al., 2021), and influence of storage at 37°C, 4°C, and −20 °C (Yuan et al., 2021). Our results showed that LPS stimuli could reduce the size and increase the number of vesicles.

In summary, we have shown that *miR-181a-5p* and *miR-23b-3p* were differentially expressed in circulating extracellular vesicles earlier than creatinine elevation in a sepsis-induced AKI rat model. These miRNAs target several molecules related to physiological and pathological conditions, especially transcription factors related to regulatory proteins involved in inflammatory processes. The difference in the expression of these miRNAs may potentially be an important tool for the early identification of sepsis-induced AKI and be useful for discriminating sepsis-induced AKI from other causes of AKI. Finally, it is worth pointing out that these miRNAs can serve as therapeutic targets in sepsis-induced AKI.

## Data Availability

The original contributions presented in the study are included in the article/Supplementary Materials, further inquiries can be directed to the corresponding author.
