# Supplementary material for: Analysis of exosome-derived microRNAs as early biomarkers of lipopolysaccharide-induced acute kidney injury in rats
Source: Front Physiol. 2022 Aug 26;13:944864. doi: 10.3389/fphys.2022.944864 (PMC9462429; doi:10.3389/fphys.2022.944864)
Supplement: Supplementary file 1 [file DataSheet1.docx]

**Table S1. Expression profile of miRNAs in LPS-treated animals compared to control.**

| **Upregulated** | **p-value** | **Downregulated** | **p-value** |
| --- | --- | --- | --- |
| *rno-let-7a-3p* | 0.330 | *rno-miR-147* | 0.093 |
| *rno-let-7e-5p* | 0.111 | *rno-miR-200a-3p* | 0.625 |
| *rno-let-7i-3p* | 0.344 | *rno-miR-200b-3p* | 0.539 |
| *rno-miR-9a-5p* | 0.353 | *rno-miR-203a-5p* | 0.359 |
| *rno-miR-16-5p* | 0.163 |  |  |
| ***rno-miR-23b-3p*** | **0.035*** |  |  |
| *rno-miR-26b-3p* | 0.342 |  |  |
| *rno-miR-30a-3p* | 0.087 |  |  |
| *rno-miR-30b-5p* | 0.053 |  |  |
| *rno-miR-30d-3p* | 0.326 |  |  |
| *rno-miR-34c-3p* | 0.320 |  |  |
| *rno-miR-106b-5p* | 0.292 |  |  |
| *rno-miR-107-3p* | 0.323 |  |  |
| *rno-miR-107-5p* | 0.347 |  |  |
| *rno-miR-122-5p* | 0.172 |  |  |
| *rno-miR-125a-3p* | 0.338 |  |  |
| *rno-miR-125a-5p* | 0.060 |  |  |
| *rno-miR-132-3p* | 0.210 |  |  |
| *rno-miR-141-3p* | 0.345 |  |  |
| *rno-miR-142-5p* | 0.333 |  |  |
| *rno-miR-146a-5p* | 0.137 |  |  |
| *rno-miR-146b-3p* | 0.330 |  |  |
| *rno-miR-146b-5p* | 0.224 |  |  |
| ***rno-miR-181a-5p*** | **0.015*** |  |  |
| *rno-miR-181d-5p* | 0.330 |  |  |
| *rno-miR-195-5p* | 0.172 |  |  |
| *rno-miR-212-3p* | 0.337 |  |  |
| *rno-miR-212-5p* | 0.334 |  |  |
| *rno-miR-221-3p* | 0.247 |  |  |
| *rno-miR-222-3p* | 0.144 |  |  |
| *rno-miR-322-5p* | 0.110 |  |  |
| *rno-miR-369-3p* | 0.348 |  |  |
| *rno-miR-369-5p* | 0.339 |  |  |
| *rno-miR-381-3p* | 0.347 |  |  |
| *rno-miR-381-5p* | 0.346 |  |  |
| *rno-miR-410-3p* | 0.325 |  |  |
| *rno-miR-429* | 0.347 |  |  |
| *rno-miR-449a-3p* | 0.321 |  |  |
| *rno-miR-497-5p* | 0.181 |  |  |
| *rno-miR-628* | 0.346 |  |  |

*p<0,05.

Table S2. Biological Process associated with *miR-23b-3p* and *miR-181a-5p* target genes according to *Enrichr*.

| **Index** | **Name** | **p-value** | **Adjusted p-value** | **Z-score** | **Combined score** |
| --- | --- | --- | --- | --- | --- |
| ***miR-181a-5p*** | | | | | |
| 1 | Regulation of nucleic acid-templated transcription (GO:1903506) | 0.000 | 0.000 | -2.13 | 37.35 |
| 2 | Regulation of cellular macromolecule biosynthetic process (GO:2000112) | 0.000 | 0.000 | -1.38 | 23.78 |
| 3 | Regulation of gene expression (GO:0010468) | 0.000 | 0.000 | -1.87 | 30.53 |
| 4 | Regulation of transcription, DNA-templated (GO:0006355) | 0.000 | 0.001 | -1.83 | 18.83 |
| 5 | Positive regulation of vesicle fusion (GO:0031340) | 0.006 | 0.121 | -3.52 | 17.83 |
| 6 | Protein localization to cytoplasmic stress granule (GO:1903608) | 0.007 | 0.121 | -2.78 | 13.73 |
| 7 | Response to leucine (GO:0043201) | 0.009 | 0.121 | -3.10 | 14.63 |
| 8 | Cellular response to leucine (GO:0071233) | 0.009 | 0.121 | -1.80 | 8.47 |
| 9 | Cellular response to leucine starvation (GO:1990253) | 0.010 | 0.121 | -1.88 | 8.69 |
| 10 | Stress granule assembly (GO:0034063) | 0.011 | 0.121 | -2.74 | 12.43 |
| ***miR-23b-3p*** | | | | | |
| 1 | Regulation of nucleic acid-templated transcription (GO:1903506) | 0.000 | 0.024 | -2.13 | 18.01 |
| 2 | Regulation of cellular macromolecule biosynthetic process (GO:2000112) | 0.000 | 0.024 | -1.38 | 11.42 |
| 3 | Cellular response to drug (GO:0035690) | 0.000 | 0.025 | -1.59 | 12.44 |
| 4 | Fatty acid oxidation (GO:0019395) | 0.001 | 0.052 | -1.82 | 12.46 |
| 5 | Regulation of gene expression (GO:0010468) | 0.002 | 0.066 | -1.87 | 11.32 |
| 6 | Positive regulation of mitochondrion organization (GO:0010822) | 0.007 | 0.066 | -1.87 | 9.39 |
| 7 | Deadenylation-independent decapping of nuclear-transcribed mRNA (GO:0031087) | 0.007 | 0.066 | -3.57 | 17.91 |
| 8 | Iron ion import (GO:0097286) | 0.007 | 0.066 | -3.13 | 15.71 |
| 9 | Regulation of relaxation of cardiac muscle (GO:1901897) | 0.007 | 0.066 | -2.99 | 15.02 |
| 10 | Positive regulation of ATP biosynthetic process (GO:2001171) | 0.007 | 0.066 | -2.73 | 13.69 |

GO, *geneontology.*

Table S3. Molecular functions associated with *miR-23b-3p* and *miR-181a-5p* target genes according to *Enrichr*.

| **Index** | **Name** | **p-value** | | **Adjusted p-value** | | **Z-score** | | **Combined score** | |
| --- | --- | --- | --- | --- | --- | --- | --- | --- | --- |
| ***miR-181a-5p*** | | | | | | | | | |
| 1 | Leucine binding (GO:0070728) | | 0.007 | | 0.075 | | -2.97 | | 14.66 |
| 2 | DNA binding (GO:0003677) | | 0.007 | | 0.075 | | -1.20 | | 5.88 |
| 3 | Eukaryotic initiation factor 4E binding (GO:0008190) | | 0.010 | | 0.075 | | -2.93 | | 13.52 |
| 4 | Annealing activity (GO:0097617) | | 0.012 | | 0.075 | | -3.34 | | 14.87 |
| 5 | RNA stem-loop binding (GO:0035613) | | 0.013 | | 0.075 | | -2.62 | | 11.48 |
| 6 | Poly(A) binding (GO:0008143) | | 0.013 | | 0.075 | | -2.21 | | 9.53 |
| 7 | Ribosomal small subunit binding (GO:0043024) | | 0.013 | | 0.075 | | -2.19 | | 9.45 |
| 8 | Poly-purine tract binding (GO:0070717) | | 0.019 | | 0.089 | | -2.43 | | 9.67 |
| 9 | mRNA 5'-UTR binding (GO:0048027) | | 0.021 | | 0.089 | | -1.94 | | 7.56 |
| 10 | ATP-dependent DNA helicase activity (GO:0004003) | | 0.026 | | 0.101 | | -1.97 | | 7.20 |
| ***miR-23b-3p*** | | | | | | | | | |
| 1 | 3',5'-cyclic-AMP phosphodiesterase activity (GO:0004115) | | 0.000 | | 0.003 | | -2.81 | | 25.79 |
| 2 | 3',5'-cyclic-nucleotide phosphodiesterase activity (GO:0004114) | | 0.000 | | 0.003 | | -2.30 | | 19.84 |
| 3 | Alpha-(1->3)-fucosyltransferase activity (GO:0046920) | | 0.009 | | 0.075 | | -3.23 | | 15.37 |
| 4 | Enoyl-CoA hydratase activity (GO:0004300) | | 0.009 | | 0.075 | | -2.97 | | 14.14 |
| 5 | cAMP binding (GO:0030552) | | 0.012 | | 0.077 | | -2.06 | | 9.07 |
| 6 | Fucosyltransferase activity (GO:0008417) | | 0.013 | | 0.077 | | -2.44 | | 10.53 |
| 7 | Ligand-deendent nuclear receptor binding (GO:0016922) | | 0.017 | | 0.085 | | -2.80 | | 11.43 |
| 8 | Cyclic nucleotide binding (GO:0030551) | | 0.021 | | 0.091 | | -1.90 | | 7.38 |
| 9 | Adenyl ribonucleotide binding (GO:0032559) | | 0.029 | | 0.111 | | -1.38 | | 4.90 |
| 10 | Peptidyl-prolyl cis-trans isomerase activity (GO:0003755) | | 0.036 | | 0.125 | | -2.10 | | 6.95 |

GO, *geneontology.*

Table S4. Cellular components associated with *miR-23b-3p* and *miR-181a-5p* target genes according to *Enrichr*.

| **Index** | **Name** | **p-value** | | **Adjusted p-value** | | **Z-score** | | **Combined score** | |
| --- | --- | --- | --- | --- | --- | --- | --- | --- | --- |
| ***miR-181a-5p*** | | | | | | | | | |
| 1 | Cytoplasmic stress granule (GO:0010494) | | 0.037 | | 0.194 | | -2.28 | | 7.52 |
| 2 | Cytosolic small ribosomal subunit (GO:0022627) | | 0.044 | | 0.194 | | -1.52 | | 4.75 |
| 3 | Ruffle membrane (GO:0032587) | | 0.048 | | 0.194 | | -2.11 | | 6.43 |
| 4 | Sall ribosomal subunit (GO:0015935) | | 0.048 | | 0.194 | | -1.50 | | 4.57 |
| 5 | Cytoplasmic vesicle membrane (GO:0030659) | | 0.048 | | 0.194 | | -1.54 | | 4.65 |
| 6 | Ribonucleoprotein granule (GO:0035770) | | 0.070 | | 0.235 | | -2.20 | | 5.83 |
| 7 | Ficolin-1-rich granule lumen (GO:1904813) | | 0.106 | | 0.246 | | -2.17 | | 4.88 |
| 8 | Cytosolic ribosome (GO:0022626) | | 0.107 | | 0.246 | | -1.29 | | 2.89 |
| 9 | Cytoplasmic vesicle lumen (GO:0060205) | | 0.111 | | 0.246 | | -1.46 | | 3.20 |
| 10 | Cytosolic part (GO:0044445) | | 0.135 | | 0.257 | | -1.54 | | 3.08 |
| ***miR-23b-3p*** | | | | | | | | | |
| 1 | P-body (GO:0000932) | | 0.002 | | 0.056 | | -1.32 | | 8.27 |
| 2 | HFE-transferrin receptor complex (GO:1990712) | | 0.009 | | 0.109 | | -3.98 | | 18.98 |
| 3 | Cytoplasmic ribonucleoprotein granule (GO:0036464) | | 0.011 | | 0.109 | | -1.51 | | 6.79 |
| 4 | DNA-directed RNA polymerase II, core complex (GO:0005665) | | 0.017 | | 0.123 | | -2.16 | | 8.82 |
| 5 | Mitochondrial proton-transporting ATP synthase complex (GO:0005753) | | 0.022 | | 0.126 | | -8.48 | | 32.52 |
| 6 | Spindle midzone (GO:0051233) | | 0.029 | | 0.140 | | -1.53 | | 5.42 |
| 7 | Mitochondrial matrix (GO:0005759) | | 0.034 | | 0.142 | | -1.15 | | 3.90 |
| 8 | Nuclear chromosome (GO:0000228) | | 0.049 | | 0.144 | | -2.12 | | 6.40 |
| 9 | Nuclear DNA-directed RNA polymerase complex (GO:0055029) | | 0.049 | | 0.144 | | -1.82 | | 5.48 |
| 10 | Perinuclear region of cytoplasm (GO:0048471) | | 0.050 | | 0.144 | | -1.72 | | 5.15 |

GO, *geneontology.*

Table S5. Cellular pathways associated with *miR-23b-3p* and *miR-181a-5p* target genes according to *Enrichr*.

| **Index** | **Name** | **p-value** | **Adjusted p-value** | **Z-score** | **Combined score** |
| --- | --- | --- | --- | --- | --- |
| ***miR-181a-5p*** | | | | | |
| 1 | miRNA regulation of p53 pathway in prostate cancer WP3982 | 0.021 | 0.086 | -2.54 | 9.77 |
| 2 | RIG-I-like Receptor Signaling WP3865 | 0.053 | 0.105 | -1.74 | 5.14 |
| 3 | Neural Crest Differentiation WP2064 | 0.087 | 0.112 | -1.10 | 2.68 |
| 4 | Angiopoietin Like Protein 8 Regulatory Pathway WP3915 | 0.112 | 0.112 | -1.09 | 2.37 |
| ***miR-23b-3p*** | | | | | |
| 1 | Phosphodiesterases in neuronal function WP4222 | 0.001 | 0.033 | -1.68 | 11.38 |
| 2 | G Protein Signaling Pathways WP35 | 0.003 | 0.050 | -1.29 | 7.31 |
| 3 | Caloric restriction and aging WP4191 | 0.008 | 0.055 | -3.12 | 15.23 |
| 4 | FTO Obesity Variant Mechanism WP3407 | 0.008 | 0.055 | -2.83 | 13.84 |
| 5 | Iron metabolism in placenta WP2007 | 0.011 | 0.055 | -2.97 | 13.30 |
| 6 | SREBF and miR33 in cholesterol and lipid homeostasis WP2011 | 0.015 | 0.055 | -2.52 | 10.56 |
| 7 | Circadian rhythm related genes WP3594 | 0.015 | 0.055 | -0.95 | 3.97 |
| 8 | Farnesoid X Receptor Pathway WP2879 | 0.018 | 0.055 | -2.50 | 10.06 |
| 9 | Mitochondrial Gene Expression WP391 | 0.018 | 0.055 | -2.49 | 10.01 |
| 10 | Transcription factor regulation in adipogenesis WP3599 | 0.021 | 0.055 | -2.44 | 9.45 |

WP, *WikiPathways.*
